# Supplementary figures and images for: Coevolutionary Analysis of Protein Subfamilies by Sequence Reweighting
Source: Entropy (Basel). 2019 Nov 16;21(11):1127. doi: 10.3390/e21111127 (PMC6992422; doi:10.3390/e21111127)

## $\alpha$ - Interface

**A**

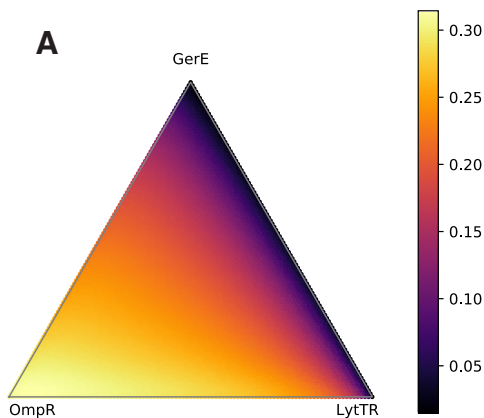

## $\beta$ - Interface

**B**

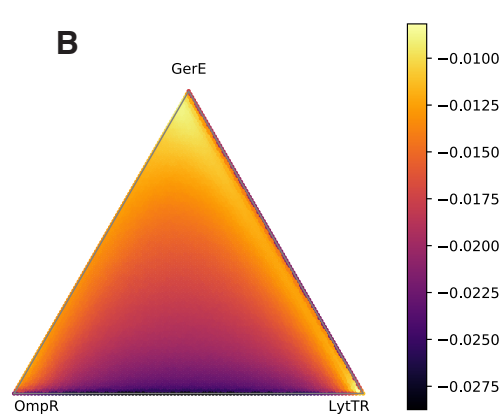

## $\gamma$ - Interface

**C**

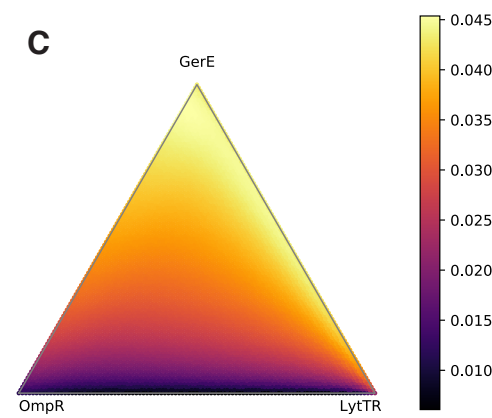

**D**

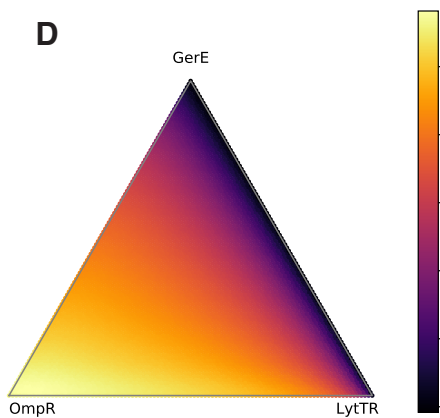

**E**

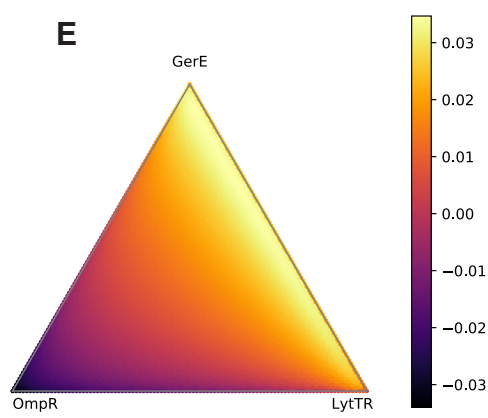

**F**

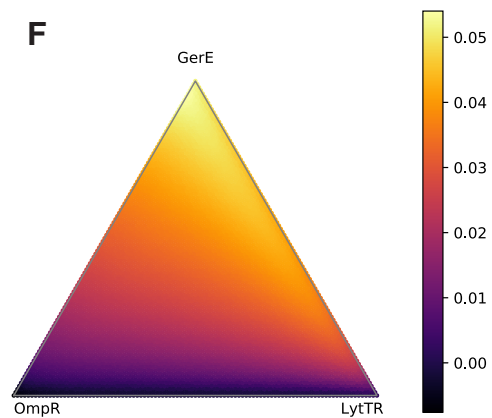

**G**

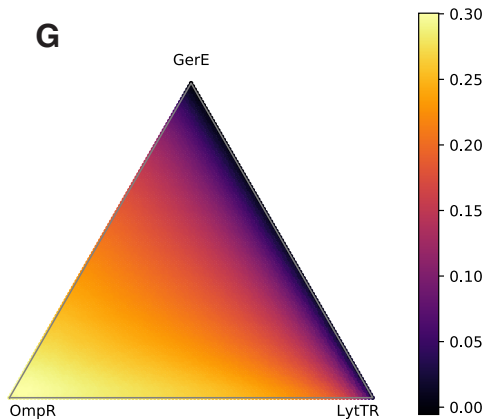

**H**

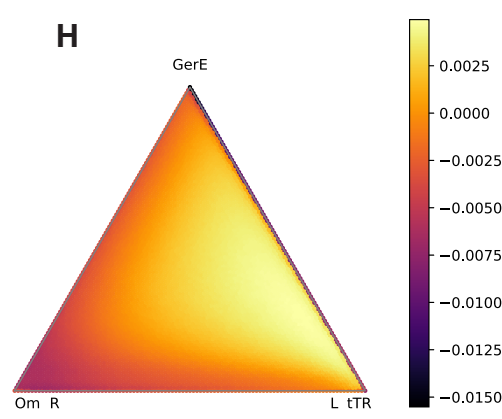

**I**

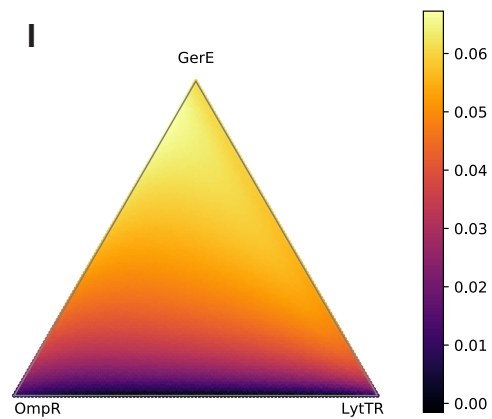

Supplement: Supplementary file 1 [file entropy-21-01127-s001.zip › FigS2.pdf]

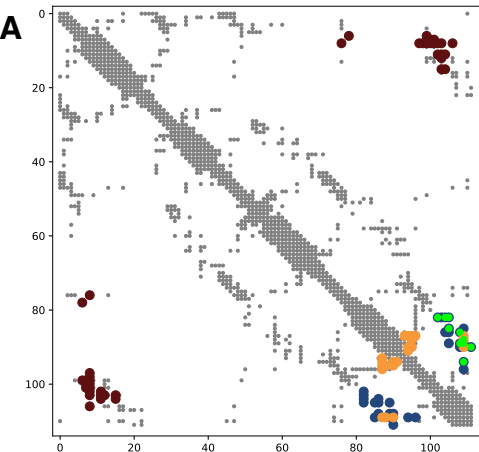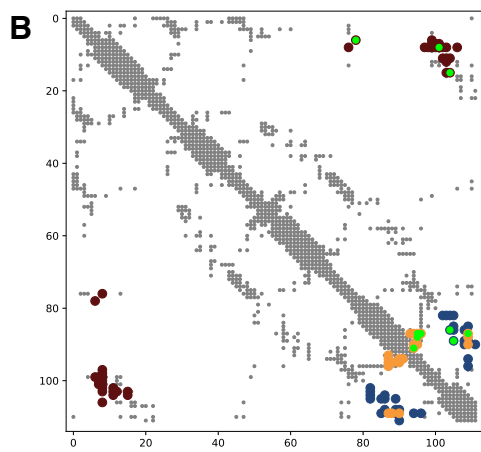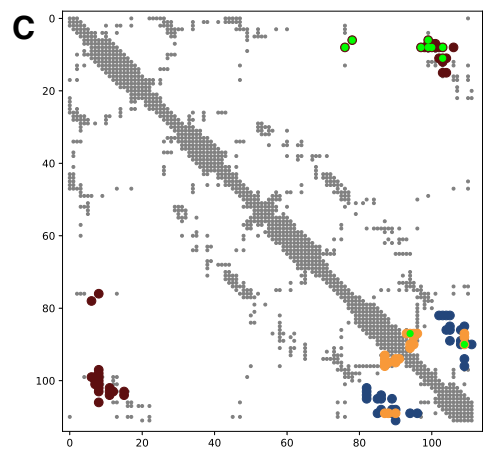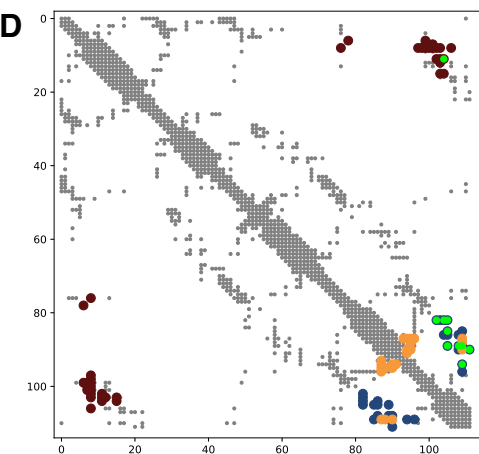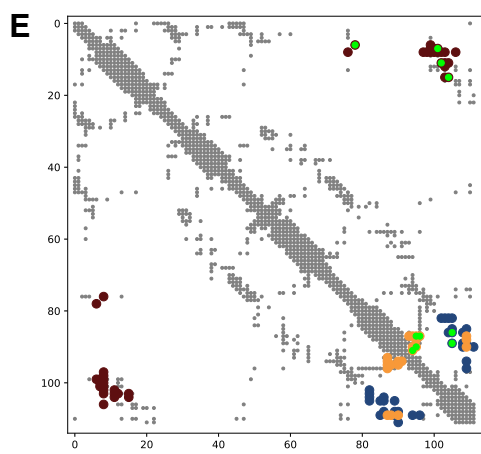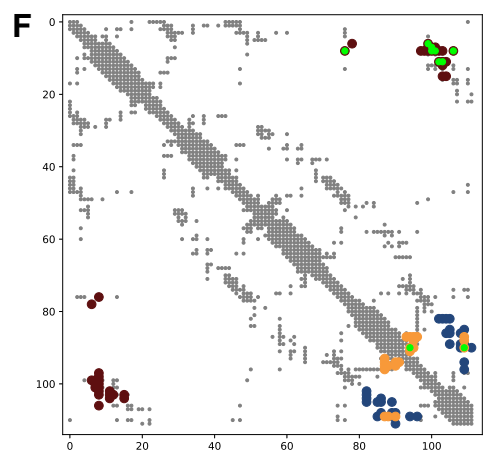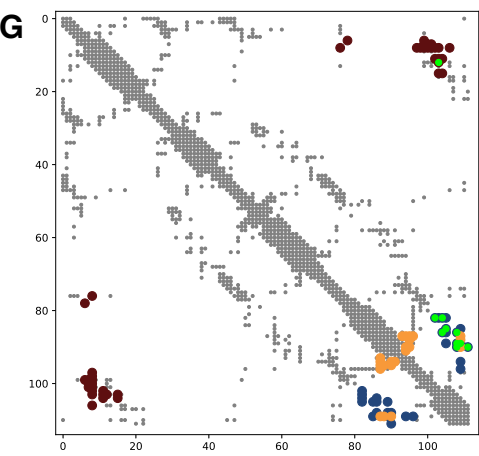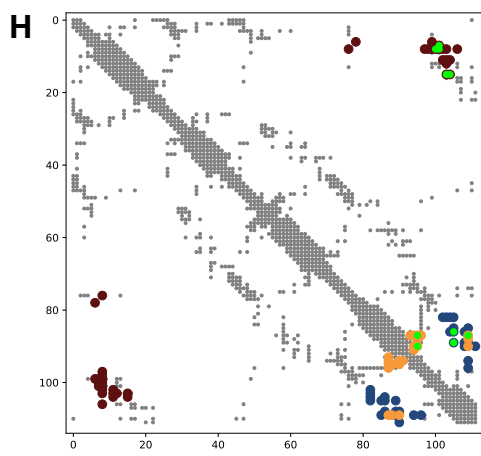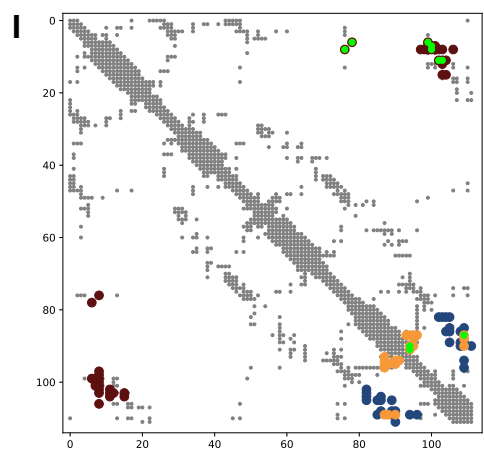

Supplement: Supplementary file 1 [file entropy-21-01127-s001.zip › FigS3.pdf]

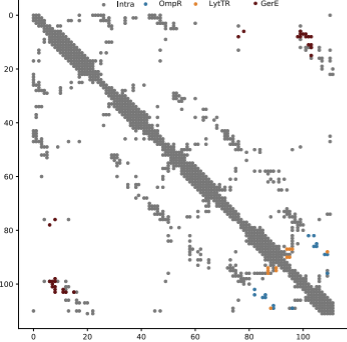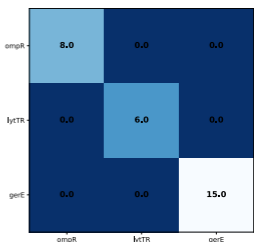

Cutoff 4Å

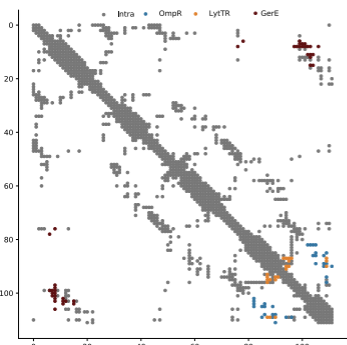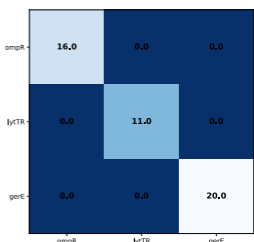

Cutoff 5Å

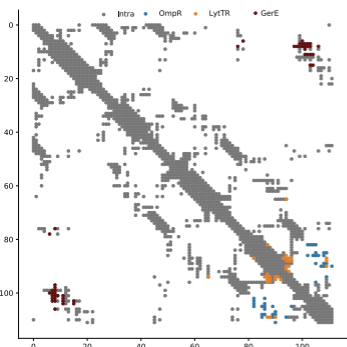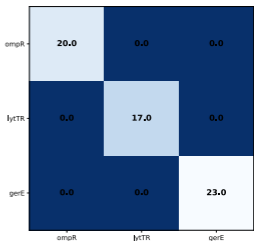

Cutoff 6Å

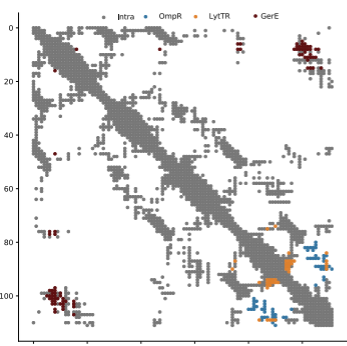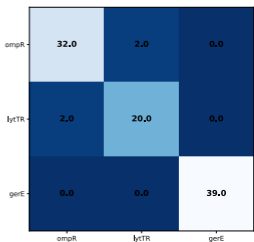

Cutoff 7Å

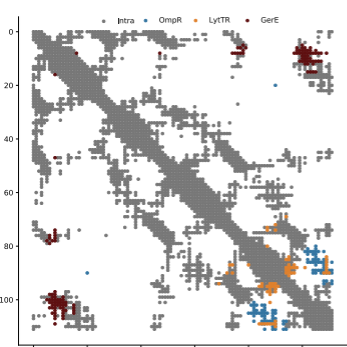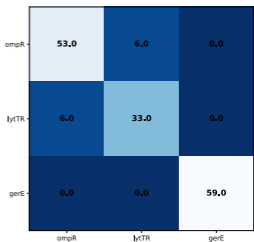

Cutoff 8Å

Supplement: Supplementary file 1 [file entropy-21-01127-s001.zip › FigS1.pdf]
